# Distinct effector functions mediated by Fc regions of bovine IgG subclasses and their interaction with Fc gamma receptors

**DOI:** 10.3389/fimmu.2023.1286903

**Published:** 2023-11-22

**Authors:** Alistair Noble, Basudev Paudyal, John C. Schwartz, William Mwangi, Danish Munir, Elma Tchilian, John A. Hammond, Simon P. Graham

**Affiliations:** The Pirbright Institute, Woking, United Kingdom

**Keywords:** cattle, IgG subclasses, Fc gamma receptor, Fc region, NK cell, monocyte, macrophage

## Abstract

Cattle possess three IgG subclasses. However, the key immune functions, including complement and NK cell activation, and enhancement of phagocytosis, are not fully described for bovine IgG1, 2 and 3. We produced chimeric monoclonal antibodies (mAbs) consisting of a defined variable region linked to the constant regions of bovine IgG1, 2 and 3, and expressed His-tagged soluble recombinant bovine Fc gamma receptors (FcγRs) IA (CD64), IIA (CD32A), III (CD16) and Fcγ2R. Functional assays using bovinized mAbs were developed. IgG1 and IgG3, but not IgG2, activated complement-dependent cytotoxicity. Only IgG1 could activate cattle NK cells to mobilize CD107a after antigen crosslinking, a surrogate assay for antibody-dependent cell cytotoxicity. Both IgG1 and IgG2 could trigger monocyte-derived macrophages to phagocytose fluorescently labelled antigen-expressing target cells. IgG3 induced only weak antibody-dependent cellular phagocytosis (ADCP). By contrast, monocytes only exhibited strong ADCP when triggered by IgG2. IgG1 bound most strongly to recombinant FcγRs IA, IIA and III, with weaker binding by IgG3 and none by IgG2, which bound exclusively to Fcγ2R. Immune complexes containing IgG1, 2 and 3 bound differentially to leukocyte subsets, with IgG2 binding strongly to neutrophils and monocytes and all subclasses binding platelets. Differential expression of the FcγRs on leukocyte subsets was demonstrated by surface staining and/or RT-qPCR of sorted cells, e.g., Fcγ2R mRNA was expressed in monocytes/macrophages, neutrophils, and platelets, potentially explaining their strong interactions with IgG2, and FcγRIII was expressed on NK cells, presumably mediating IgG1-dependent NK cell activation. These data reveal differences in bovine IgG subclass functionality, which do not correspond to those described in humans, mice or pigs, which is relevant to the study of these IgG subclasses in vaccine and therapeutic antibody development.

## 1 Introduction

**Figure 1:**
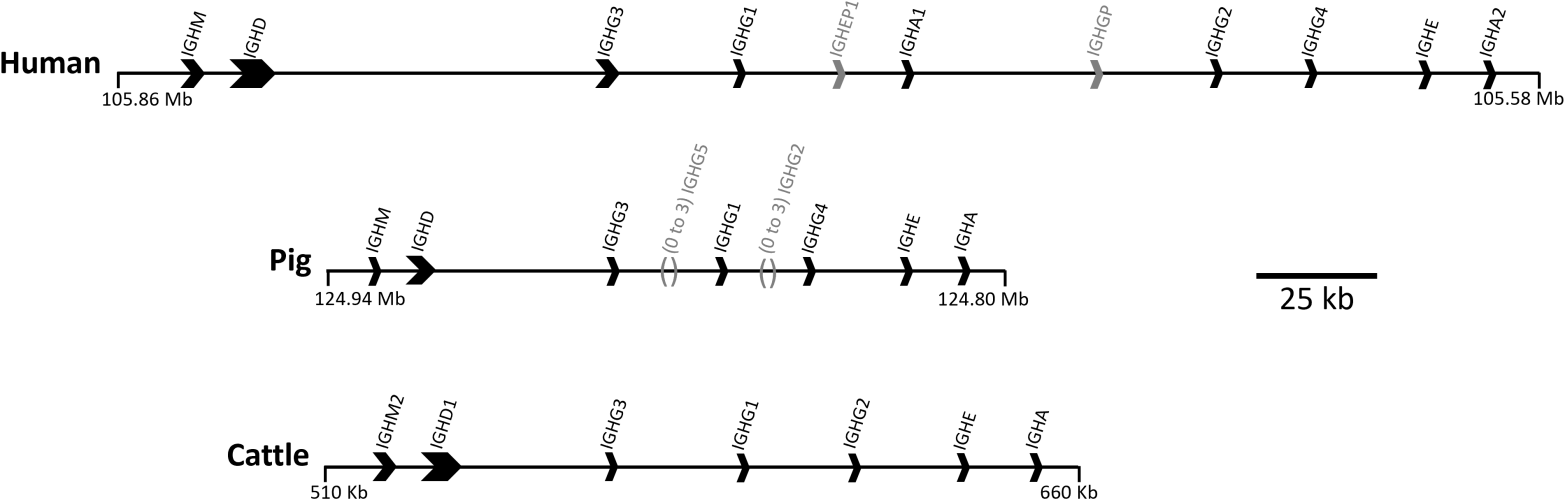
Organization of the IGH constant region in the human, pig, and cattle genomes. Arrows point in the direction of transcription. Pseudogenes *IGHEP1* and *IGHGP* in human are shown in gray. A minimal gene configuration is shown for the pig, which is variable in gene content, specifically for *IGHG2* and *IGHG5* which are shown in gray along with their relative location. The pig haplotype used to generate this schematic also contains *IGHG2b*, but for simplicity of visualization this was removed. Coordinates shown are for the GenBank accessions for human (GRCh38.p14; Chr 14), pig (TPI_Babraham_pig_v1; Chr 7), and cattle: CM000676.2, CM062304.1, KT723008, respectively. The gene organization for cattle is based on a bacterial artificial chromosome assembly (https://pubmed.ncbi.nlm.nih.gov/27053761/).

The major circulating antibody (Ab) in mammals, IgG, is of great importance to immune function in health and disease. IgG subclasses differ in structure, serum half-life, abundance, interaction with FcγRs and effector functions, including complement activation and enhancement of phagocytosis and NK cell activity (1). IgG subclasses evolved after speciation, so there is no orthology between subclasses in different species (2). Hence, although IgG subclasses in humans and mice are relatively well characterized, little is known about IgG subclass effector functions in other species. There are four IgG subclasses in humans, rats and mice, while cattle, goats and sheep have three, and pigs have nine (**Figure 1**) (3–6).

The effector functions of IgG subclasses are mediated by the Fc region of their heavy chains and do not affect the ability of Ab to bind epitopes and, therefore, to neutralize pathogens such as viruses by blocking entry to target cells (7). Complement-dependent cytotoxicity (CDC) is mediated by IgG binding to antigen on a target cell triggering the classical complement pathway via C1q, and is an important mechanism via which antibodies can kill tumor or virally infected cells (8). In certain viral infections, such as HIV and SARS-CoV-2, the Fc-mediated functions of IgG are of significant clinical importance (9, 10). The ability of certain IgG subclasses to enhance cellular phagocytosis (ADCP) is key to the use of therapeutic mAbs in cancer treatment (11). IgG binding to FcγRIII on human NK cells allows these cytotoxic lymphocytes to be activated by high-affinity antibodies (12), providing an important link between innate and acquired immunity. Additionally, Fc-mediated functions can play important roles in immunopathology, especially where immune complexes are formed and complement activated (13).

**Figure 2:**
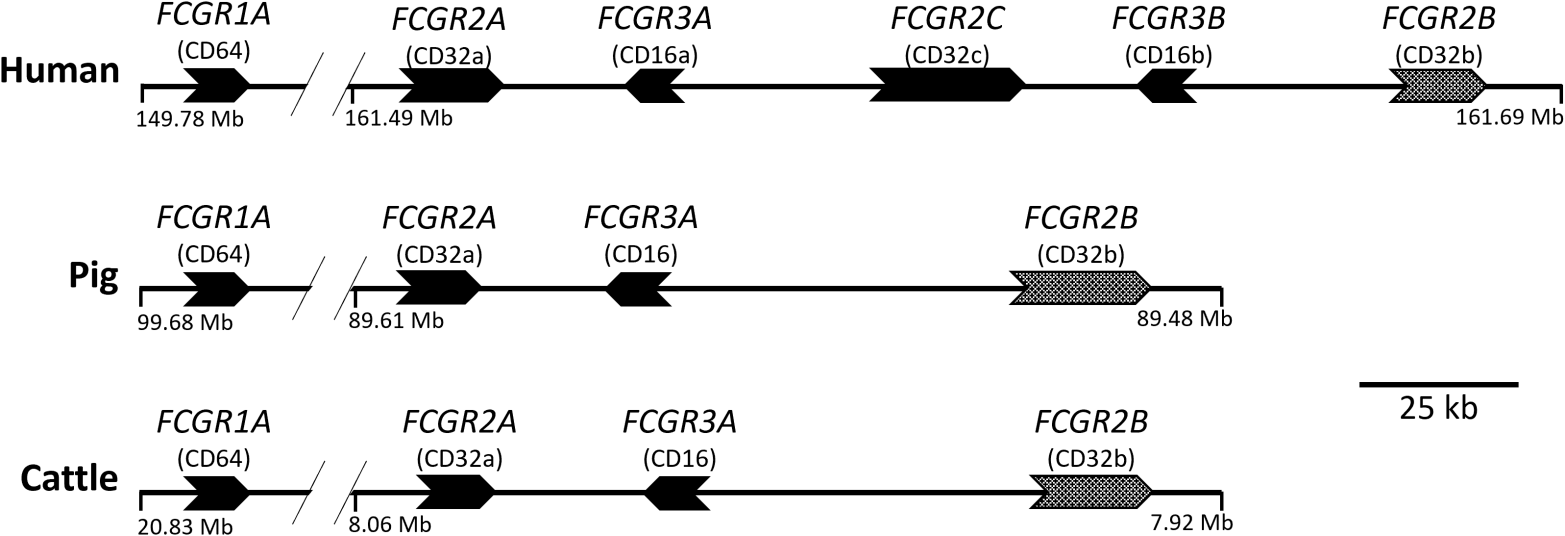
Organization of the FcγR genes in the human, pig, and cattle genomes. Arrows point in the direction of transcription. *FCGR2B* is alternatively shaded to indicate its different signaling characteristics. *FCGR1A* and *FCGR2A* are separated by approximately 10 to 13 Mb. The intergenic regions shown do not contain additional FcgR gene fragments in either pigs or cattle, as investigated using both BLAST (https://pubmed.ncbi.nlm.nih.gov/2231712/) and CD-Search (https://pubmed.ncbi.nlm.nih.gov/15215404/). *FCG2R* is not shown as it is found elsewhere in the genome and is unique to bovids, such as cattle. Coordinates shown are for the GenBank accessions for Human (GRCh38.p13; Chr 1), Pig (TPI_Babraham_pig_v1; Chr 4), and Cattle (ARS-UCD1.2; Chr 3): CM000663.2, CM062301.1, and CM008170.2, respectively.

We recently characterized the effector functions of the porcine IgG subclasses; utilizing recombinant subclass-switched mAbs specific for influenza A virus hemagglutinin (IAV HA) as a model system to directly compare Fc functions (6). Here we have used the same approach to characterize the functions of IgG subclasses of cattle, a species of enormous agricultural and economic importance. We have mapped IgG subclass functions against the expression of cattle FcγRs (**Figure 2**), including the unique Fcγ2R receptor (14, 15), on different leukocyte populations. We determined IgG subclass binding to these populations and measured IgG subclass binding properties to the recombinant FcγRs. Our data reveal previously unrecognized differences in function, which expands knowledge of IgG subclass diversity in mammals.

## 2 Materials and methods

### 2.1 Generation of recombinant chimeric monoclonal antibodies expressing bovine Fc regions

Bovinized antibodies consisting of the Fab region from a previously characterized porcine mAb (pb27) specific for IAV HA from (pH1N1) pdm09 (16), combined with the bovine Fc regions from IgG1, 2 and 3 subclasses, were generated. This allowed us to utilize assay systems for the Fc function we previously developed for the porcine IgG subclasses (6). Expression vectors were made based on pNeoSec backbone as previously described (16). Constant regions for bovine IgG1 (GenBank accession # S82409.1), IgG2 (GenBank accession # KT761528.1), IgG3 (GenBank accession # U63638.1) and lambda light chain (LC-λ; GenBank accession # HQ456934.1) were obtained from public databases and ordered as synthetic gBlock Gene Fragments (Integrated DNA Technologies/IDT) with extensions allowing directional cloning. The resulting vectors, pNeoSec-BovFc-IgG1, pNeoSec-BovFc-IgG2, pNeoSec-BovFc-IgG3 and pNeoSec-BovLC-λ, encode the μ-phosphatase leader sequence and contain the antibody V-region cloning site in frame to a downstream constant region for expression as recombinant mAbs.

VDJ heavy chain and VJ light chain coding gBlocks Gene Fragments of mAb pb27 were directionally cloned by in-fusion ClonExpress II One Step Cloning Kit (Vazyme) into corresponding vectors linearized with *Kpn*I and *Pst*I and transformed into chemically competent *E. coli* Stellar cells (Takara Bio Inc.) according to manufacturer’s protocols. Plasmids encoding chimeric pb27-BovFc-IgG1, -BovFc-IgG2, and -BovFc-IgG3 were co-transfected with chimeric pb27-BovLC-λ into Expi293F™ cells (ThermoFisher) at 1 mL scale in 50 mL mini bioreactors (Corning) according to Protein Production UK’s standard Expi293™ transfection protocol and Gibco Expi293™ Expression System User Guide (ThermoFisher Scientific Publication Number: MAN0007814). Briefly, Expi293™ cells were grown at 37°C, 8% carbon dioxide and on the day of transfection they were at a cell density of 2 × 10^6^/mL. 0.5 μg of the plasmid DNA construct for the heavy chain and 0.5 μg of the light chain construct were mixed in 0.1 mL Opti-MEM (ThermoFisher) containing 5.3 μg of polyethylenimine PEI 40K MAX (Polysciences) and this mixture was added to the Expi293™ cells at 2x10^6^/in 1 mL. After 18 hours the following enhancers (all Merck) were added: 17 µl of 50 mg/mL valproic acid, 7 µl 100 mg/mL sodium propionate and 18 µl 45% glucose. Cell-free supernatants were harvested after 3-days incubation. The presence of bovine IgG was determined by Western blot analysis under non-reducing conditions using sheep anti-bovine IgG-HRP (BioRad) with ECL Prime Reagent substrate (Cytiva). Selected mAbs were scaled up for expression at 300 mL followed by purification on the ÄKTA pure 25 by 5 mL Protein G chromatography (Cytiva), and then buffer exchanged into Dulbecco’s PBS without calcium and magnesium (**Supplementary Figure 1**). The mg/mL concentration of the purified antibodies was determined by A_280_/1.4 and the antibodies were diluted to 1 mg/mL in PBS (**Supplementary Table 1**).

Control bovine IgG1, 2 and 3 mAbs were generated as described above, except the variable regions were taken from a bovine mAb, B4, specific for bovine respiratory syncytial virus (BRSV) fusion (F) protein (17) (**Supplementary Figure 2**, **Supplementary Table 1**).

### 2.2 Generation of recombinant soluble FcγRs

Bovine FcγRs were expressed as soluble proteins using extracellular domains (ECDs) fused to Avi tag and a six Histidine (6xHis) tag at the C-terminus (**Supplementary Figure 3**). The predicted ECDs of bovine FcγRI (FCGR1A, Chr 3: 20802002-20811359), FcγRIIA (FCGR2A, Chr 3: 8035689-8044512), FcγRIII (FCGR3A, Chr 3: 8000299-8008181), and Fcγ2R (FCG2R, Chr 18: 62945907-62948192) were manually annotated within the cattle reference genome assembly, ARS-UCD1.2 (18), using Artemis (v17.0.1) (19) and available Iso-Seq data (BioProject accession #: PRJNA386670) (20). These annotated sequences were further confirmed to be identical to GenBank accessions NM_174538.2, NM_001109806.1, NM_001077402.1, and AAI40641.1, respectively. Expression constructs for FcγRI, IIA and III were designed and constructed by Twist Bioscience using pTwist CMV vector system with bovFcγRI and FcγRIIA fused to human CD5 leader peptide, whereas FcγRIII utilized the native leader peptide. Fcγ2R sequence, codon optimized and synthesized as gBlock Gene Fragments (IDT), was cloned into a pNeoSec-derived expression vector containing μ-phosphatase leader sequence by In-Fusion cloning (ClonExpress II One Step Cloning Kit, Vazyme) after linearizing the vectors with KpnI (5’) and ScaI (3’). The DNA fragments contained a 15 bp extension on both ends corresponding to the expression vector, allowing In-Fusion cloning and in-frame insertion from the leader sequence to generate expression constructs as described above.

FcγRs were transiently transfected into Expi293F™ cells as described above. Following 3 days of incubation, the cell culture supernatant was harvested, purified by IMAC HisTrap column (Cytiva) followed by buffer exchange with PBS as described above (**Supplementary Figure 4**, **Supplementary Table 2**).

### 2.3 Antigen-binding assays

Indirect ELISAs were performed using 96-well Nunc MaxiSorp plates (ThermoFisher) coated with 10 µg/mL recombinant soluble HA from IAV pH1N1 A/England/195/2009 (kindly provided by Professor Alain Townsend, University of Oxford, UK) (21) followed by titrated recombinant bovinized mAbs, then biotinylated rabbit anti-bovine IgG(H+L) (ThermoFisher) and streptavidin-HRP (Bio-Rad). Assays were performed in PBS with 0.05% Tween 20 and developed using TMB substrate (Merck).

### 2.4 FcγR-binding assays

For FcγR:mAb binding ELISAs, recombinant FcγRs were coated at 10 µg/mL prior to addition of titrated antibodies. Binding was detected using biotinylated HA (prepared using ThermoFisher EZ-Link™ Sulfo-NHS-Biotinylation Kit, according to manufacturer’s instructions) at 1 µg/mL, followed by streptavidin-HRP. To assess binding of mAbs to cell-associated antigen, Madin–Darby canine kidney–2,6-sialyltransferase cells stably expressing HA from IAV pH1N1 A/Eng/195/2009 (MDCK-HA), provided by Professor Alain Townsend, were used. Cultured MDCK-HA cells were washed, stained with Zombie Aqua™ Fixable Viability Kit (BioLegend) and incubated with serially diluted mAbs in PBS with 0.1% BSA. Cells were washed and incubated with rabbit anti-bovine IgG(H+L)-biotin (ThermoFisher) followed by streptavidin-Brilliant Violet 421 (BV421; BioLegend). All antibodies used in this study are detailed in **Supplementary Table 3**. In some cases, mAbs were fluorochrome labelled in-house using Lightning-Link kits (Abcam).

### 2.5 Bovine leukocyte isolation

250 mL heparinized blood was collected from six healthy animals aged between 7 and 18 months, housed at the Centre for Dairy Research, University of Reading under housing and procedures approved by the local Animal Welfare and Ethical Review Bodies and conducted in accordance with the Animals (Scientific Procedures) Act, UK. Leukocytes were prepared by diluting anti-coagulated cattle blood 1 in 4 in PBS, pelleting cells at 400 × g for 10 min, then lysing the cell pellet with 50 mL RBC Lysis Buffer (BioLegend) for 5 min and washing 3 times with PBS. Peripheral blood mononuclear cells (PBMC) were prepared from 200 mL heparinized blood diluted 1 in 3 in PBS and centrifuged over Histopaque 1.083 (Merck) at 1000 × g for 40 min. PBMC were collected from the interface and washed 3 times in PBS.

### 2.6 FcγR binding analysis

To assess binding of bovine IgG subclasses to leukocytes, mAbs were incubated at 5 µg/mL in XVIVO15 medium (Scientific Laboratory Supplies) with an equimolar concentration of purified IAV swine A/swine/England/1353/2009 (pH1N1) (12.5 pM) for 15 min to generate immune complexes (ICs). Cryopreserved leukocytes were thawed, washed in PBS, and added at 2 × 10^5^ per well in 96-well U-bottom plates, to immune complexes at 4°C. After 1 h, cells were washed, stained with Zombie Aqua™ stain and immune complex binding detected with anti-bovine IgG1/2/3-PerCPCy5.5 (**Supplementary Table 3**). Binding to NK cells, monocytes, platelets, and neutrophils was determined by co-staining with NKp46-PE, CD14-APC-Cy7, CD41/61-PE-Cy7 and CD11b-Alexa Fluor 647 mAbs respectively (**Supplementary Table 3**).

### 2.7 Biolayer interferometry

Biolayer interferometry (BLI) was carried out on an Octet R8 instrument (Sartorius) to characterize interaction kinetics between mAbs and recombinant FcγRs. All steps were performed at 30°C with the microplate agitated at 1,000 rpm and data acquisition rate of 5.0 Hz. Kinetics buffer (Sartorius) was used to dilute reagents, to hydrate NTA biosensors (Sartorius) and to establish baselines, except for Fcγ2R experiments, where Superblock buffer (Thermo Fisher Scientific) was used instead to prevent non-specific binding of IgG2 to unloaded sensors. Recombinant FcγRs were immobilized onto biosensors at 1.5 nM for 300 s (200 nM for Fcγ2R). The probes were then blocked for 300 s with 0.2% casein followed by 300 s re-equilibration. The association step in mAbs at 100, 50, 25, 12.5, 6.25, 3.13 and 0 nM lasted for 120 s followed by dissociation for 300 s. Data were aligned to the association step, interstep corrected to association and Savitsky Golay correction was applied. Data were fitted using a 1:1 stoichiometry model and R^2^ values > 0.98 were achieved. Data analysis was conducted using the Octet Analysis Software v12.2.1.23 (Sartorius).

### 2.8 Complement-dependent cytotoxicity assay

The ability of mAbs to activate the complement cascade was performed using rabbit low-tox-H complement (Cedarlane Laboratories). This was pre-adsorbed on MDCK-HA cells for 1 h at 4°C prior to use. MDCK-HA cells in XVIVO15 serum-free medium were cultured at 3 × 10^4^ cells/well in U-well plates with serially diluted mAbs and pre-absorbed rabbit complement diluted 1/32 in XVIVO15. After 1 h at 37°C cells were centrifuged at 400 × g for 5 min and 100 μL of supernatant transferred to a flat bottom plate, to which 100 µL of LDH-substrate was added to measure the lactate dehydrogenase (LDH) released from lysed cells (LDH-Cytox Assay kit, BioLegend). The reaction was stopped after 30 min at room temperature (RT) and A_490_ determined using a GloMax plate reader (Promega). Complement activation was expressed as % lysis of target cells compared to maximum lysis induced by the addition of 2% Triton-X100. In some experiments, bovine plasma, prepared from PBMC separation, as above, was pre-absorbed against MDCK-HA cells and added at a range of dilutions to target cells in the presence or absence of rabbit complement and mAbs.

### 2.9 Antibody-dependent cellular phagocytosis assay

Monocytes were enriched from washed PBMC by labelling cells with anti-CD14-PE mAb, washing in PBS, labelling with anti-PE microbeads (50 µL/10^8^ cells), and performing positive selection on a MACS LS column (both Miltenyi Biotec) according to manufacturer’s instructions. Monocytes (purity > 95%) were used directly in ADCP assays or incubated with 20 ng/mL of recombinant bovine M-CSF (Cambridge Bioscience) at 2 × 10^6^ per mL in RPMI-1640 medium (ThermoFisher) with 10% FBS (Life Science Production) for 7 days to differentiate them into macrophages. Cultures were performed in ultra-low adherence microplates (Costar) to allow recovery of macrophages. MDCK-HA target cells were fluorescently labelled with CellTrace Far Red (ThermoFisher) following the manufacturer’s instructions and incubated with serially diluted mAbs for 15 min at RT in a U-well polypropylene plate. Monocytes or macrophages were labelled with CellTrace Violet (ThermoFisher) and added at an effector to target ratio of 4:1, in XVIVO15 medium at 5 ×10^4^ cells/well. The plates were centrifuged at 150 × g for 2 min and incubated at 37°C for 3 h. Cells were washed and labelled with Zombie Green Fixable Viability Kit (BioLegend) and analyzed by flow cytometry as described above. The proportion of live monocytes/macrophages that contained target cell material (CellTrace Violet^+^ CellTrace Far Red^+^) was calculated.

### 2.10 NK-cell degranulation assay

NK cells were enriched from freshly isolated PBMC by labelling with anti-NKp46-PE mAb (25 µL/10^8^ cells) followed by anti-PE microbeads (Miltenyi Biotec, 50 µL/10^8^ cells). Two rounds of positive selection were then performed on an LD MACS™ depletion column, to capture the weakly labelled NKp-46^+^ cells. The proportion of NKp46^+^ cells in PBMC ranged from 0.2 ­ 5.0% in different animals and they were enriched to between 40 - 80% by this procedure. NK enriched fractions were stimulated for 2 days in XVIVO15 serum-free medium at 2 × 10^6^ per mL with recombinant human (rh) IL-2 (10 ng/mL), rhIL-12 (50 ng/mL) and rhIL-18 (50 ng/mL) (all BioLegend), which are known to activate bovine cells (22). After washing in PBS, degranulation of differentiated NK cells was detected using surface expression of CD107a during a 5 h stimulation as a readout. ELISA plates were coated overnight at 4°C with 100 µg/mL recombinant HA protein in carbonate-bicarbonate buffer. After washing in PBS, mAbs at 10 µg/mL were added in PBS with 0.1% BSA for 1 h at RT. NK cells were washed and added at 5 × 10^4^ cells/well in XVIVO15 with anti-CD107a-APC (a gift from Dr Timothy Connelley, The Roslin Institute, University of Edinburgh) (manuscript in preparation) (1/500) and monensin (3 µM, BioLegend) and incubated for 5 h at 37°C. Cells were washed and stained with Zombie Aqua Fixable Viability Kit, anti-NKp46-PE, anti-CD3-PerCPCy5.5 and anti-CD40-FITC (**Supplementary Table 1**). The cells were washed, fixed, and analyzed by flow cytometry as described above.

### 2.11 Flow cytometry and cell sorting

Flow cytometry analysis was performed using a MACSQuant Analyzer 10 flow cytometer (Miltenyi Biotec) and FCS files were analyzed using FlowJo 10 software (BD Biosciences). For cell sorting, leukocytes were stained as above and sorted into platelets, monocytes, and neutrophils using FSC/SSC and markers as above, plus lymphocytes using FSC/SSC alone, using a FACS Aria U3 cell sorter with FACSDiva**™** software (BD Biosciences). 488, 633 and 405 nm lasers were used, with BP 530/30, 586/42, 780/60, 660/20, 780/60 and 525/50 filters used to detect FITC, PE, PE-Cy7, Alexa-647, APC-Cy7 and Zombie Aqua**™** stains respectively. In some experiments a Bigfoot Spectral Cell Sorter (ThermoFisher) was used with 9-laser setup and Sasquatch software. PBMC were enriched for NK cells as described above and sorted to obtain NK cells (NKp46^+^CD40^-^CD3^-^) and non-NK (NKp46^-^CD40^-^CD3^-^) fractions. Typically, ~3 x 10^5^ cells were collected into FBS, centrifuged, and resuspended in RLT buffer (Qiagen).

### 2.12 Quantitative reverse-transcribed PCR (qRT-PCR) for FcγRs in leukocytes and platelets

RNA from sorted cell populations or monocyte-derived macrophages (as described above) was purified using the RNeasy Micro Kit (Qiagen). RNA was quantified using the QuantiFluor RNA system and GloMax Discover plate reader (both Promega). Five ng RNA was reverse transcribed using the LunaScript RT SuperMix kit (New England BioLabs). qPCR reactions were performed using the Luna Universal qPCR Master Mix (New England BioLabs) using 0.5 µL cDNA and 0.5 µL 10 µM primers in a 20 µL reaction volume. Primers used are listed in **Supplementary Table 4**. qPCR was performed in a QuantStudio 5 Real Time PCR System (Thermo Fisher Scientific) and analyzed using QuantStudio Design and Analysis Software v1.5.1. Forty cycles of PCR were performed with 15 s at 95°C, 30 s at 52°C and 30 s at 72°C. Standard curves (10^2^ – 10^8^ copies per sample) were constructed for each target using PCR amplicons gel-purified and quantified by spectrophotometry (23). Cycle threshold values were determined in duplicate and interpolated against the standard curves to determine the number of mRNA copies per ng total RNA for each sample.

### 2.13 Statistical analysis

Pooled data from independent experiments are presented as mean ± SEM and statistical differences calculated by ANOVA with multiple comparisons using GraphPad Prism 9 software, as indicated. In all figures significant differences are shown as *: p<0.05; **: p<0.01; ***: p<0.001; ****: p<0.0001.

## 3 Results

### 3.1 Bovine IgG subclass switched chimeric mAbs display unaltered antigen binding

**Figure 3:**
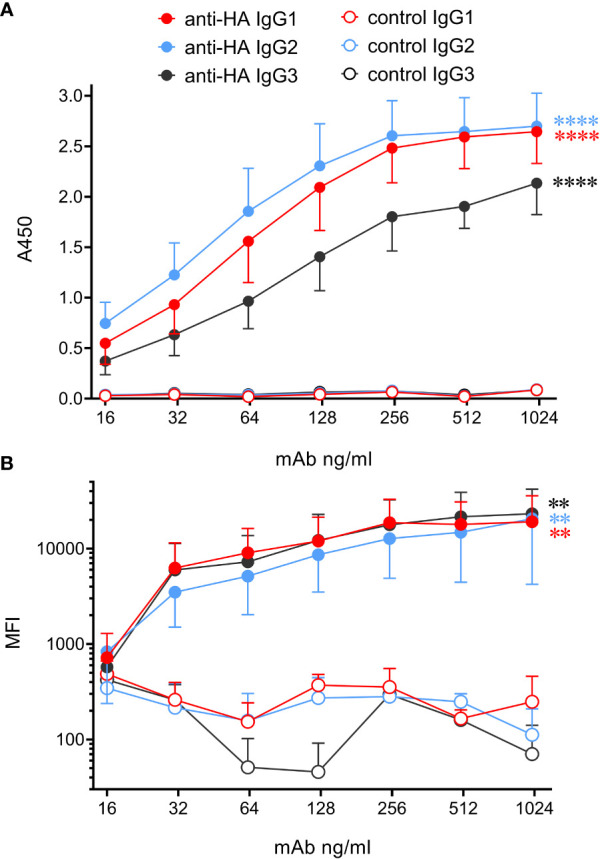
Bovinized recombinant mAbs bind to immobilized or cell-associated antigen with similar affinity. **(A)** ELISA data using HA-coated plates and titrated mAbs followed by biotinylated rabbit anti-bovine IgG(H+L) and streptavidin-HRP. Data are means ± SEM from three independent experiments. **(B)** MDCK-HA antigen-expressing cells were harvested and stained with serially diluted mAbs, washed, and incubated with rabbit anti-bovine IgG(H+L)-biotin, followed by streptavidin-Brilliant Violet 421. Flow cytometric median fluorescence intensity (MFI) of staining was obtained. Means ± SEM from three independent experiments. Statistics refer to two-way ANOVA comparisons of anti-HA mAbs to their respective control mAbs. **p<0.01; ****p<0.0001.

Analysis of high-quality genomic data indicated that cattle express only 3 IgG subclasses – IgG1, 2 and 3. Gene segments encoding Fc regions of each were spliced to the variable region of the porcine IAV HA-specific mAb pb27 (16), or to that of the BRSV F protein specific mAb, B4 (17). The corresponding light chains were also constructed and recombinant mAbs expressed and purified (**Supplementary Figures 1**, **2**). To determine whether the engineering of the IgG subclass influenced the binding to antigen, we measured mAb binding to immobilized pH1N1 HA by ELISA and to MDCK-HA cells by flow cytometry (**Figure 3**). All three IgG subclasses bound to plastic immobilized HA and cell-associated HA, and control mAbs showed no binding. The binding of plate-bound HA by IgG1 and IgG2 did not differ significantly (p>0.05) but both bound more strongly than IgG3 (p<0.05). However, no significant differences were observed in the binding of the subclasses to MDCK-HA cells (p>0.05). Thus, as found in our porcine study (6), switching Fc regions of pb27 mAb had a minimal effect on the affinity of antigen binding.

### 3.2 Binding of bovine IgG subclasses to recombinant FcγRs

**Figure 4:**
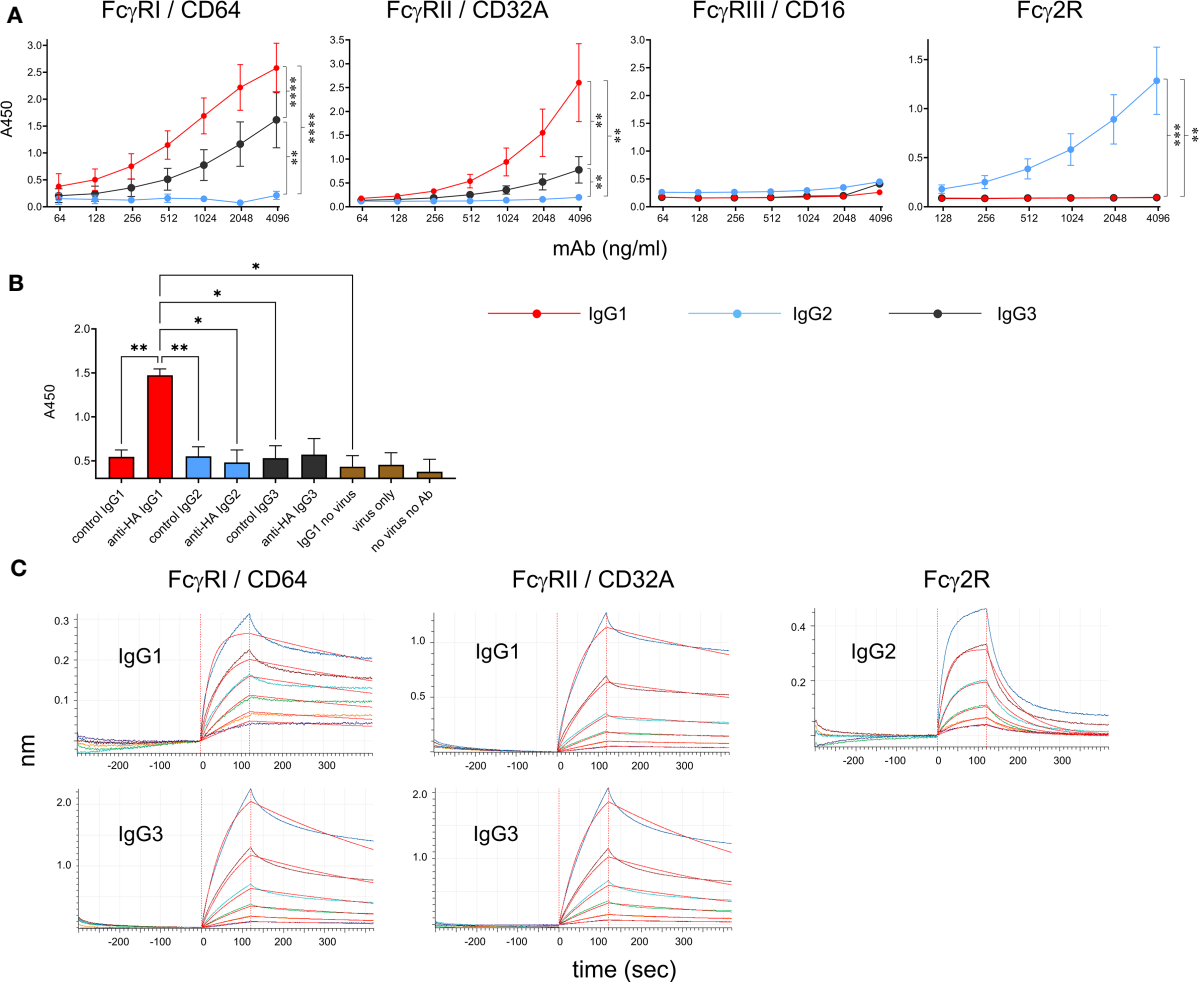
Binding properties of recombinant cattle FcγRs. **(A)** Binding of recombinant IgG1, IgG2 and IgG3 to recombinant FcγRs, detected by ELISA. Anti-HA mAbs were titrated onto immobilized soluble receptors and detected with HA-biotin. Data are means ± SEM from three independent experiments. Results from a two-way ANOVA analysis with multiple comparisons are indicated. **(B)** Binding of recombinant FcγRIII/CD16 to immune complexes of pH1N1 virus and mAbs, detected by ELISA. Immune complexes were detected in the ELISA using anti-bovine IgG (H&L)-biotin and streptavidin-HRP. Means ± SEM from 3 independent assays are shown with one-way ANOVA analysis. **(C)** BLI analysis of binding kinetics between positive receptor-ligand interactions identified in **(A)** Graphs show equilibration, association in IgGs and dissociation in buffer alone. Colored lines show binding at 100, 50 25, 12.5, 6.25 and 3.13 nM IgG and red lines show curve fitting by Octet analysis software. Results of kinetic analysis are shown in **Table 1** and are representative of three independent experiments. *p<0.05; **p<0.01; ***p<0.001; ****p<0.0001.

Recombinant soluble bovine FcγRI (CD64, the high-affinity receptor), FcγRIIA (CD32A), FcγRIII (CD16, low-affinity receptor) and Fcγ2R (a receptor unique to cattle, goats and sheep (14, 24), were tested for their ability to bind to the bovinized anti-HA IgG subclass mAbs using ELISA (**Figure 4A**). FcγRs were immobilized onto plates and IgG subclass binding was detected using HA-biotin. Results revealed that IgG1 and IgG3 bound FcγRI and FcγRIIA but not FcγRIII or Fcγ2R. IgG1 had a higher affinity for both receptors. By contrast, IgG2 only bound to Fcγ2R. Since human FcγRIII is known to bind multimeric but not monomeric IgG, we tested the ability of immune complexes (IC) to bind immobilized FcγRs (**Figure 4B**). ICs containing the pH1N1 virus and IgG1 were able to bind FcγRIII, but those containing IgG2 or IgG3 were not.

To determine kinetic constants for the detectable interactions between receptors and monomeric IgG, we performed kinetic binding assays using BLI (**Figure 4C**). FcγRs were loaded onto nickel-containing biosensors (which bind His-tagged protein), blocked with casein, re-equilibrated and then dipped into wells containing IgG followed by buffer. Association and dissociation curves were determined for 120 and 300 sec, respectively. Kd values, association and dissociation constants (on rate/off rate) were calculated (**Table 1**). The values were similar to those reported for the human FcγRI and FcγRII (25). The Fcγ2R receptor showed a nanomolar affinity for IgG2.

**Table 1:** Kinetic binding constants for IgG subclass binding to recombinant FcγRs:.

RECEPTOR	LIGAND	Kd (M)	Kd error	K_ass_ ^i^ (ms^-1^)	K_ass_ error	K_dis_^ii^ (s^-1^)	K_dis_ error
FcγRI	IgG1	2.83 x 10^-9^	2.02 x 10^-11^	4.26 x 10^5^	3.53 x 10^3^	1.02 x 10^-3^	6.07 x 10^-6^
FcγRI	IgG3	1.04 x 10^-8^	7.69 x 10^-11^	1.56 x 10^5^	1.02 x 10^3^	1.61 x 10^-3^	5.55 x 10^-6^
FcγRIIA	IgG1	7.36 x 10^-9^	4.78 x 10^-11^	1.70 x 10^5^	9.15 x 10^2^	1.25 x 10^-3^	4.55 x 10^-6^
FcγRIIA	IgG3	2.02 x 10^-8^	2.08 x 10^-10^	5.65 x 10^4^	4.38 x 10^2^	1.14 x 10^-3^	7.72 x 10^-6^
Fcγ2R	IgG2	1.92 x 10^-8^	2.18 x 10^-10^	7.04 x 10^5^	7.69 x 10^3^	1.36 x 10^-2^	4.14 x 10^-5^

i Rate constant of association.

^ii^Rate constant of dissociation.

### 3.3 Binding of IgG subclasses to monocytes, macrophages, platelets, neutrophils, and natural killer cells

**Figure 5:**
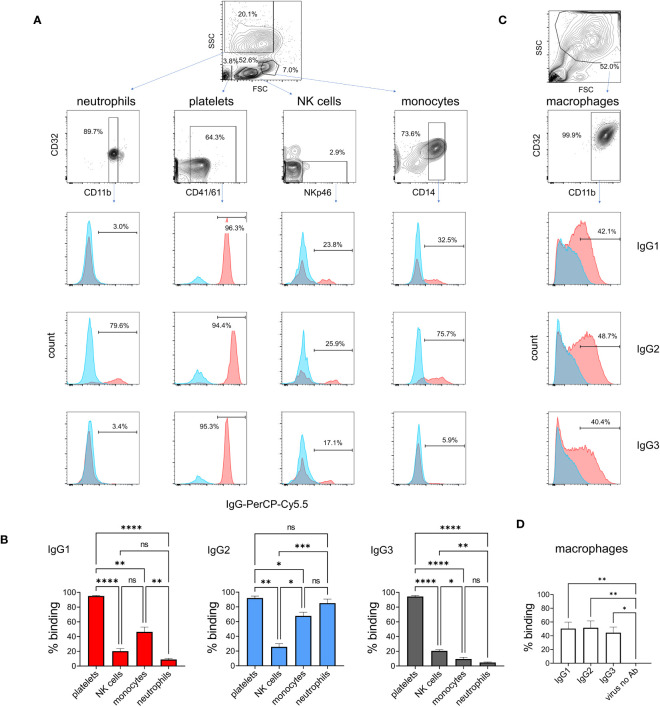
Binding of IgG1, 2 and 3 to naturally expressed FcγRs on leukocytes. Leukocytes were prepared by lysis of whole blood and stained with immune complexes of pH1N1 virus and IgG1, 2 or 3 anti-HA mAbs. Neutrophils, platelets, NK cells and monocytes were gated according to forward scatter (FSC) and side scatter (SSC) properties and cell surface markers as shown. **(A)** Binding profiles revealed by anti-IgG - red histogram shows immune complex binding and blue histogram shows binding of control (monomeric) mAb. **(B)** Mean data ± SEM from five independent experiments showing % positive binding by flow cytometry. **(C, D)** Identical staining on cultured macrophages using gating as shown. Results from one-way ANOVA analysis with multiple comparisons are indicated. ns, not significant; *p<0.05; **p<0.01; ***p<0.001; ****p<0.0001.

To determine IgG subclass binding to immune cells known to express FcγRs, we prepared leukocytes and platelets, and incubated them at 4°C with ICs of pH1N1 and mAbs (**Figures 5A, B**). Cell subsets were then gated during flow cytometric analysis using a combination of forward/side scatter characteristics and staining with surface markers. IC binding was detected using an anti-bovine IgG 814448 mAb that binds equally to IgG1, IgG2 and IgG3 (data not shown). The data revealed that all platelets bound ICs most strongly, irrespective of IgG subclass. A subset of NK cells bound IgG1, IgG2 and IgG3 ICs, without significant differences (p<0.05). By contrast, neutrophils, and to a lesser extent monocytes, preferentially bound IgG2-containing ICs (p<0.01 and p<0.05, respectively). Monocyte-derived macrophages, showing a typical forward/side scatter profile and CD32A^+^CD11b^+^ phenotype, bound IgG1, IgG2 and IgG3 ICs equally (**Figures 5C, D**).

### 3.4 Expression of FcγRs on bovine leukocyte subsets

**Figure 6:**
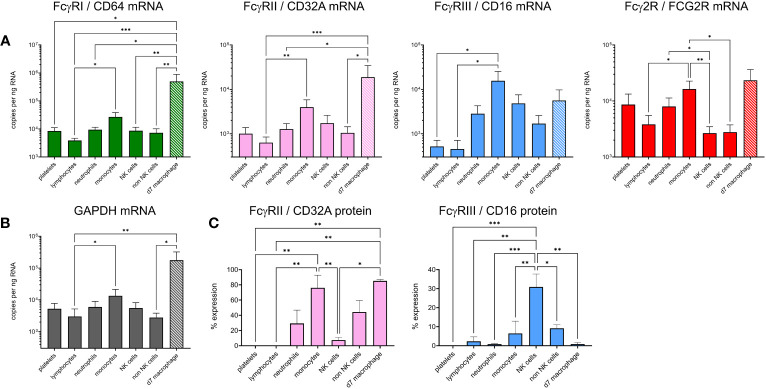
Expression of FcγRs on cattle leukocyte subsets and macrophages. **(A)** Freshly isolated leukocytes were sorted into subsets by flow cytometry and compared with unsorted, cultured monocyte-derived macrophages (day 7 of M-CSF-culture – d7 macrophage, hatched bars). The gating of subsets was as in **Figure 5**. RT-qPCR was used to assess FcγR mRNA levels. Data are means + SEM from five independent experiments, normalized to total RNA. **(B)** Expression of reference gene GAPDH, assessed as in **(A)**. **(C)** Cell surface protein expression of CD32A and CD16 using fluorochrome-labelled mAbs and flow cytometry. Cells were gated as in **(A)** but not sorted. Results from one-way ANOVA analysis with multiple comparisons are indicated. *p<0.05; **p<0.01; ***p<0.001.

To explain the IC binding pattern on leukocyte subsets, we performed cell sorting and RT-qPCR for the FcγRs (**Figures 6A, B**). Detection mAbs were only available for bovine FcγRIII and FcγRIIA, so flow cytometric detection of these receptors at the protein level was also performed (**Figure 6C**). mRNA levels of sorted freshly prepared leukocytes were also compared with cultured M-CSF-induced macrophages. Since mRNAs for reference genes were not equally expressed between subsets, we normalized the data to total RNA in **Figure 6A** and show GAPDH reference gene levels in **Figure 6B**. The FCGR1A expression data revealed high level expression in macrophages and significantly higher expression in monocytes than lymphocytes. Macrophages also expressed higher levels of FCGR2A and FCG2R than other subsets, potentially explaining their ability to bind all three IgG subclasses. Monocytes expressed FCG2R and FCGR3A mRNA, but negligible FcγRIII protein at the cell surface possibly reflecting different protein turnover or post–transcriptional–translational regulatory mechanisms. By contrast, FcγRIIA was expressed at protein and mRNA levels. NK cells, as expected, expressed FcγRIII. Control populations – lymphocytes or non-NK cells from each sort, expressed low levels of all FcγRs as expected. Although not significant compared to control populations, neutrophils expressed higher levels of FCG2R and FCGR2A mRNA, with a proportion of cells expressing measurable FcγRIIA protein, possibly reflecting their strong IgG2 binding.

### 3.5 Cattle IgG subclasses and complement-dependent cytotoxicity

**Figure 7:**
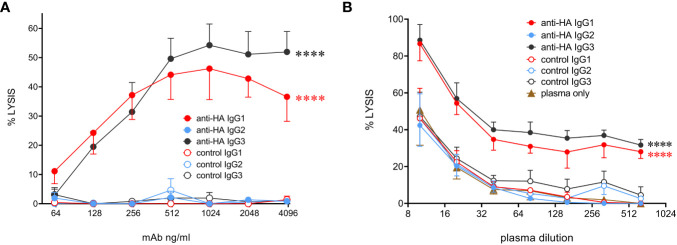
Complement-dependent cytotoxicity **(CDC)** mediated by the cattle IgG subclasses. **(A)** MDCK-HA target cells were incubated with anti-HA or control mAbs as indicated at a range of concentrations before addition of rabbit complement and detection of cell lysis by LDH release. Means ± SEM from four independent experiments. Statistics refer to two-way ANOVA comparisons of anti-HA IgG1&3 vs IgG2 mAbs. **(B)** Lysis was induced as in A but with addition of titrated bovine plasma, as well as indicated mAbs at 1 µg/mL. Means ± SEM from three independent experiments. Statistics refer to two-way ANOVA comparisons of anti-HA IgG1&3 vs IgG2 mAbs. ****p<0.0001.

To determine the ability of bovine IgG subclasses to trigger the classical complement pathway, we used MDCK-HA cells that constitutively express the HA antigen on their surface. Purified rabbit complement was used to lyse mAb-coated MDCK-HA target cells (**Figure 7A**). The data showed that IgG1 and IgG3 mediated similar levels of target cell lysis, but IgG2 could not activate complement. To check that the use of rabbit complement was not affecting the results due to the possible absence of a critical bovine plasma component required for the full pathway to be triggered, we performed the assay in the presence of both titrated bovine plasma and rabbit complement, with or without a fixed mAb concentration (**Figure 7B**). This revealed that bovine plasma could enhance target cell lysis at high concentrations, and this was further enhanced by anti-HA IgG1 and IgG3 mAbs, but not IgG2. This was demonstrated by the rightward shift in the dose-response curves in the presence of these antibodies. Thus, IgG2 could not activate complement even in the presence of bovine plasma.

### 3.6 Cattle IgG subclasses and antibody-dependent cellular phagocytosis

**Figure 8:**
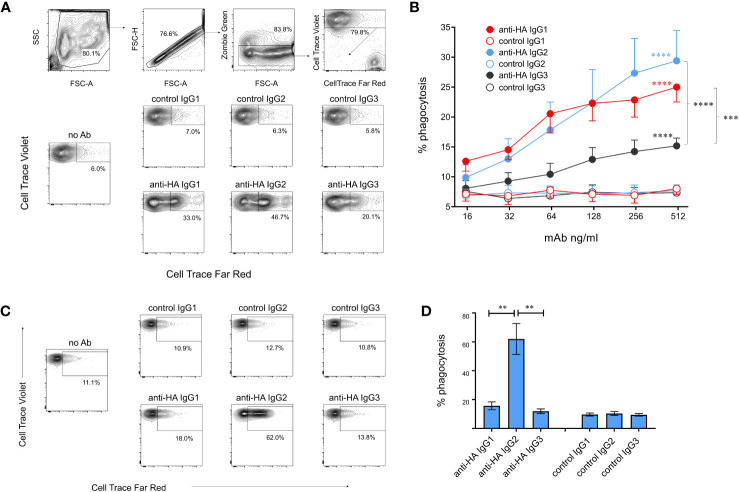
Antibody-dependent cellular phagocytosis (ADCP) mediated by cattle IgG subclasses. **(A)** ADCP mediated by macrophages was determined using MDCK-HA target cells labelled with Cell Trace Far Red and recombinant mAbs as indicated. Cultured macrophages labelled with Cell Trace Violet were gated as shown and acquisition of target cell material assessed after a 3-hour co-culture. **(B)** Means ± SEM from four independent experiments with titrated mAbs. Statistics refer to two-way ANOVA with multiple comparisons of anti-HA vs controls mAbs, or comparisons vs IgG3 as indicated; experiment as in **(A)**. **(C)** ADCP mediated by freshly isolated monocytes, detected as in **(A, D)** Means ± SEM from four independent experiments, as in **(C)**. **p<0.01; ***p<0.001; ****p<0.0001.

We next evaluated ADCP, in which phagocytic cells are triggered via FcγRs to ingest antibody-coated target cells. Monocyte-derived macrophages were cultured with MDCK-HA cells coated with mAbs and phagocytosis was assessed by measuring uptake of fluorescent-labelled target cell material by flow cytometry (**Figure 8B**). ADCP was calculated from the percentage of viable singlet macrophages that contained target cell material. Dose-dependent phagocytosis was detected with all three IgG subclasses (**Figure 8B**), but ADCP induced by IgG3 was significantly weaker than that induced by IgG1 and 2. We also determined ADCP mediated by freshly isolated monocytes as a comparison (**Figures 8C, D**). IgG2 induced extremely high levels of monocyte ADCP, significantly greater than IgG1 and IgG3 mAbs and control mAbs. IgG1 mediated weak and IgG3 almost no ADCP in monocytes, with neither being significantly different from control mAbs (p>0.05).

### 3.7 Cattle IgG subclasses and NK cell degranulation

**Figure 9:**
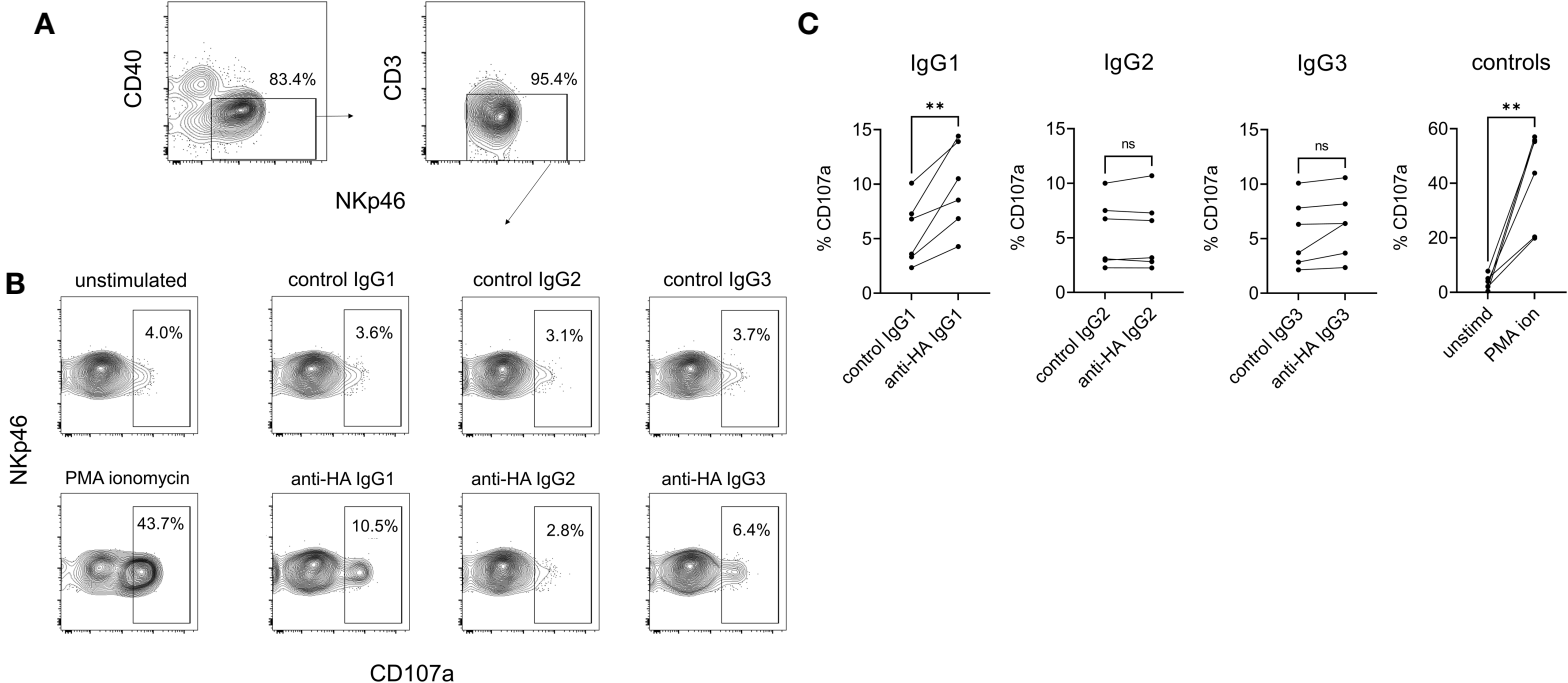
NK cell degranulation mediated by cattle IgG subclasses. NK cells were enriched from PBMC by positive selection of NKp46-expressing cells and activated by IL-2/IL-12/IL-18. **(A)** Gating of NK cells analyzed, based on CD40, CD3 and NKp46. **(B)** Surface mobilization of CD107a as an indicator of NK activation induced by plate-bound HA + recombinant mAbs, over a 5-hour stimulation. PMA/ionomycin was the positive control stimulation. **(C)** Pooled data from six independent experiments, analyzed by one-way ANOVA with multiple comparisons. ns, not significant; **p<0.01.

NK cell degranulation was assessed using surface mobilization of CD107a as an indicator of NK activation (26). To enhance responses NK-enriched PBMC were first stimulated with IL-2, IL-12, and IL-18 to induce NK cell differentiation (27). Washed effector cells were then restimulated with plate-bound antigen/mAb-coated plates or with PMA/ionomycin as a positive control stimulation. The results showed that IgG1 but not IgG2 or IgG3 was able to stimulate NK cell degranulation (**Figure 9**), which corresponded to the ability of only IgG1 ICs to bind FcγRIII (**Figure 4B**). To confirm that the activity was entirely dependent on the Fc regions of the Abs and unaffected by their specificity, we performed a cross-over experiment where the NK cells were activated with plate-bound BRSV F-protein, the target antigen for the negative control Abs. The data (**Supplementary Figure 5**) showed NK cell activation by anti-BRSV-F-IgG1 but not by anti-HA bovinized IgG1, suggesting that IgG effector functions are unrelated to Ag specificity and our observations could be generally applied.

## 4 Discussion

The function of antibodies in immune responses involves not only antigen binding by the Fab region but also the interaction of Fc regions with complement or FcγRs. FcγRs are transmembrane receptors expressed on various cell types and can be involved in phagocytosis, cytotoxic degranulation, release of cytokines and active oxygen metabolites from inflammatory cells, clearance of immune complexes and regulation of B cell antibody production. Cattle possess three Cγ genes and five FcγRs: FcγRI, FcγRIIA, FcγRIIB, FcγRIII, FcRn, which are all orthologous to human receptors (28–31), and the Fcγ2R receptor which is encoded within the leukocyte receptor complex (LRC) by a leukocyte immunoglobulin-like receptor (LILR) gene, FCG2R, and is only found in bovids (e.g., cattle, sheep and goats) (24).

Although limited data exists on IgG1 and IgG2 (32), data on IgG3 effector functions in cattle have not been described until now. The expression patterns and function of the FcγRs, particularly the bovid-specific Fcγ2R receptor, have also remained obscure. Here we have elucidated the key functions of the IgG subclasses and mapped them to FcγR expression. The specialist functions of FcRn and FcγRIIB were not studied and would require further assay development. It has been speculated that Fcγ2R evolved to bind the truncated hinge region of IgG2 (14, 33). We show here that IgG2 binds exclusively to Fcγ2R and allows IgG2 to bind strongly to monocytes and neutrophils, mediating ADCP in the case of monocytes. Other Fc-mediated functions were mediated exclusively by IgG1 (NK cell activation) or by IgG1 and IgG3 (complement activation). Thus, although we demonstrate similar Fc-related functions to those described in humans, mice, and pigs, they are mediated by different IgG subclasses which show no correspondence with other species.

To further understand the significance of these findings in terms of immunity to pathogens, it is necessary to relate IgG subclasses to classes of immune response induced by T cell subsets. Little data exists in cattle, although it is thought that IgG1 is Th2-associated and IgG2 is Th1-associated (34, 35). Immune deviation is thought to be critical in BRSV and *Mycobacterium bovis* infections of cattle, where Th2-associated responses are thought to result in pathology and poor pathogen control (36). IgG1, but not IgG2, is transferred across the mammary gland epithelium into colostrum for the protection of neonates (37). Further work is required to elucidate the role of IgG3 and the relationship of subclasses to the full complement of T cell subsets, interactions in the germinal center and possible regulatory roles for the abundant γδ T cell subset in cattle (38). This is hampered by the lack of IgG3-specific mAbs. Regulation of IgG subclass switching is dependent on the initial priming conditions but also on timing of antigen exposure. For example, repeated SARS-CoV-2 mRNA vaccination in humans results in a switch from IgG1 and IgG3 towards IgG4, a non-inflammatory subclass which does not activate complement or mediate ADCP (39). In cattle, we could not designate a clearly non-inflammatory IgG subclass and further studies are needed to understand class switch regulation.

The distinct binding affinities of the cattle FcγRs for IgG subclasses were in a similar range to human IgG : FcγRs and corresponded to the high affinity (FcγRI), lower affinity (FcγRIIA) and lowest affinity (FcγRIII) designations of other species. The differences in Kd were largely dependent on the faster association rates of FcγRI compared to FcγRIIA and of IgG1 vs IgG3. The Kd for IgG2 binding to Fcγ2R was similar to FcγRIIA but was related to a faster association rate and also faster dissociation. The distinct binding affinities, coupled with patterns of intracellular signaling from FcγRs, are responsible for the distinct biological functions associated with antibody production. No binding to FcγRIII (CD16) could be detected unless IgG1 immune complexes were used to increase avidity, explaining the exclusive ability of IgG1 to mediate NK cell activation, presumably via FcγRIII. IC binding to NK cells in mixed leukocyte preparations, however, revealed binding of a subset of NK cells to all IgG subclasses. This discrepancy may be due to the enrichment and culture of NK cells with cytokines, required for the NK cell degranulation assay, resulting in a purer and/or more homogeneous population of NK cells. Although FcγRIIA was expressed on monocytes, IgG1 induced minimal ADCP in these cells. We could therefore speculate that monocyte phagocytosis, mediated mostly by the IgG2:Fcγ2R, interaction could play a prominent role in pathogen clearance. Macrophages expressed high levels of all FcγRs except FcγRIII, at least at the mRNA level, although comparison with other cells is hampered by the fact that we used cultured, monocyte-derived macrophages. These cells bound, and mediated ADCP, with all three subclasses. Neutrophils, like monocytes, appeared to interact most strongly with IgG2, presumably via Fcγ2R expression. Further work is needed to fully elucidate the roles of the subclasses, particularly IgG3 and roles for IgGs in peripheral tissues, tumor biology and autoimmunity.

Our study highlights the understudied role for platelets in FcγR-mediated immune functions. In cattle, we show that platelets are the main source of FcγRs in the blood, considering their abundance, and we know from other species that they can mediate immune functions including release of soluble CD40L and RANTES (40) and enhance IL-1β secretion from macrophages and neutrophils (41). Thus, the strong binding of all IgG subclasses to platelets could allow early IgG secretion to trigger cellular recruitment to sites of antigenic exposure and release of further inflammatory mediators, or to mediate immune adherence, a process whereby platelet binding to pathogens mediates rapid clearance from the blood (42). These functions could be triggered by all three IgG subclasses.

The effector functions for bovine IgG subclasses are summarized in **Table 2**, alongside their binding profiles to FcγRs and leukocytes. These characteristics could be considered in the design of future vaccination strategies for bovid species, since goat and sheep IgG1, IgG2, and IgG3 are orthologous to those in cattle, and they also have Fcγ2R (24). The assays described here could be used to define Fc function in sera from infected or vaccinated animals. Additionally, therapeutic antibodies could be designed to mediate specific combinations of neutralization and Fc-mediated functions for specific pathologies.

**Table 2:** Binding and functional properties of cattle IgG subclasses.

IgG SUB-CLASS	MACRO-PHAGES	MONO-CYTES	NK CELLS	NEUTRO-PHILS	PLATELETS	BINDING TO FcγRI	BINDING TO FcγRIIA	BINDING TO FcγRIII	BINDING TO Fcγ2R	CDC	NK ACTIV-ATION	ADCP-MACRO-PHAGE	ADCP-MONO-CYTE
IgG1	+++	+	+	–	+++	+++	+++	+	–	+++	+	+++	+
IgG2	+++	+++	+	+++	+++	–	–	–	++	–	–	+++	+++
IgG3	++	+/-	+	–	+++	++	++	–	–	+++	–	+	–

+, present; ++, strongly present; +++, very strongly present; +/-, weakly present; *p<0.05; **p<0.01; ***p<0.001; ****p<0.0001.

## Data availability statement

The original contributions presented in the study are included in the article/**Supplementary Material**. Further inquiries can be directed to the corresponding author.

## Ethics statement

The animal study was approved by The Pirbright Institute and University of Reading Animal Welfare and Ethical Review Bodies. The study was conducted in accordance with the local legislation and institutional requirements.

## Author contributions

AN: Data curation, Formal Analysis, Investigation, Methodology, Writing – original draft. BP: Methodology, Writing – review & editing. JS: Data curation, Investigation, Methodology, Writing – review & editing. WM: Investigation, Methodology, Writing – review & editing. DM: Investigation, Methodology, Writing – review & editing. ET: Conceptualization, Methodology, Writing – review & editing. JH: Conceptualization, Funding acquisition, Methodology, Project administration, Resources, Writing – review & editing. SPG: Conceptualization, Methodology, Supervision, Writing – original draft.
