# Supplementary material for: Distinct effector functions mediated by Fc regions of bovine IgG subclasses and their interaction with Fc gamma receptors
Source: Front Immunol. 2023 Nov 22;14:1286903. doi: 10.3389/fimmu.2023.1286903 (PMC10702552; doi:10.3389/fimmu.2023.1286903)
Supplement: Supplementary file 1 [file DataSheet_1.pdf]

Supplementary Material

Distinct effector functions mediated by Fc regions of bovine IgG subclasses and their interaction with Fc gamma receptors

Alistair Noble, Basudev Paudyal, John C. Schwartz, William Mwangi, Danish Munir, Elma Tchilian, John A. Hammond, Simon P. Graham

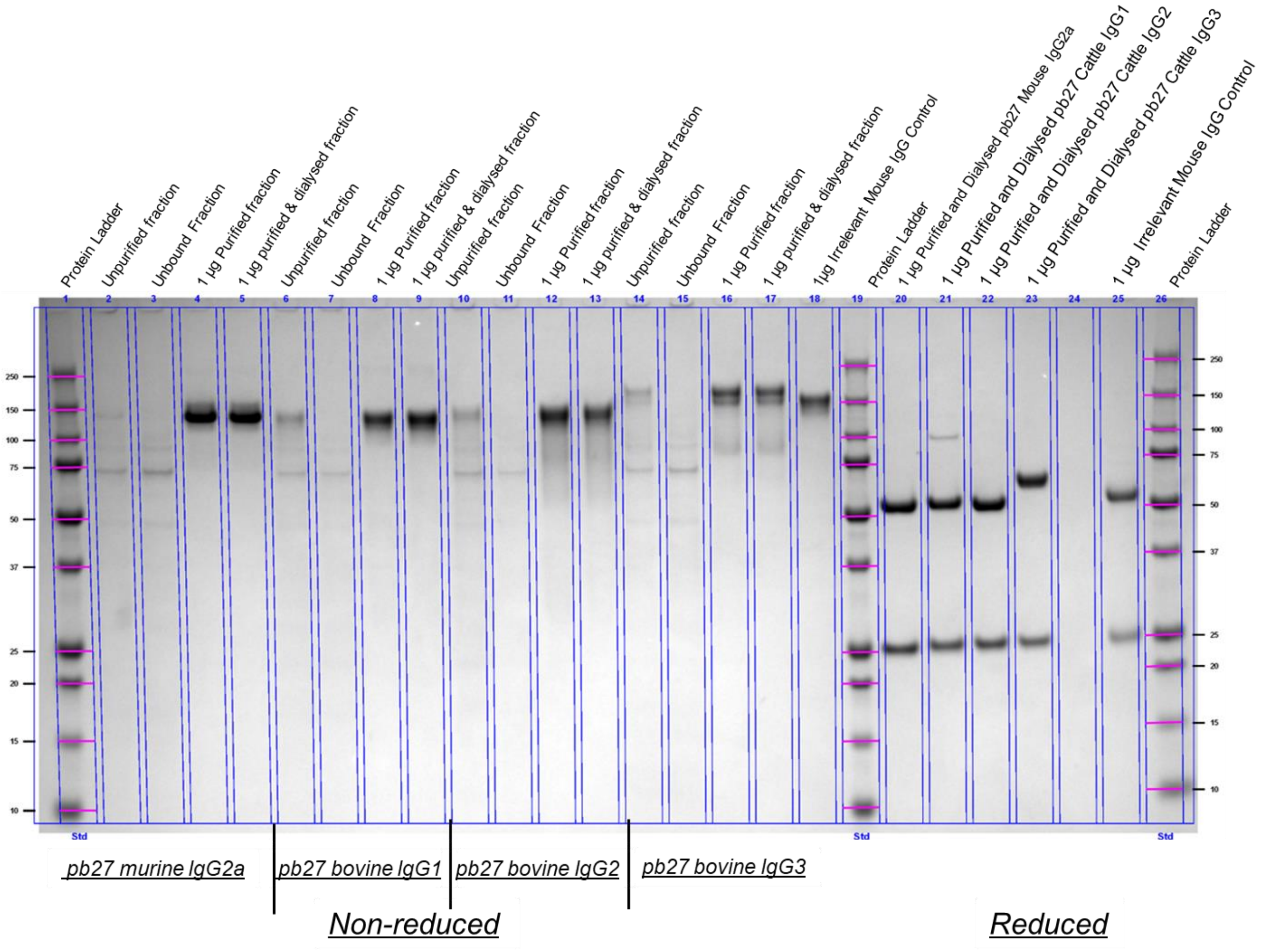

Supplementary Figure 1. SDS-PAGE (4-12% Bis-Tris gel) analysis of purified recombinant chimeric pb27 mAb expressing bovine IgG1, IgG2 and IgG3 and murine IgG2a Fc regions.

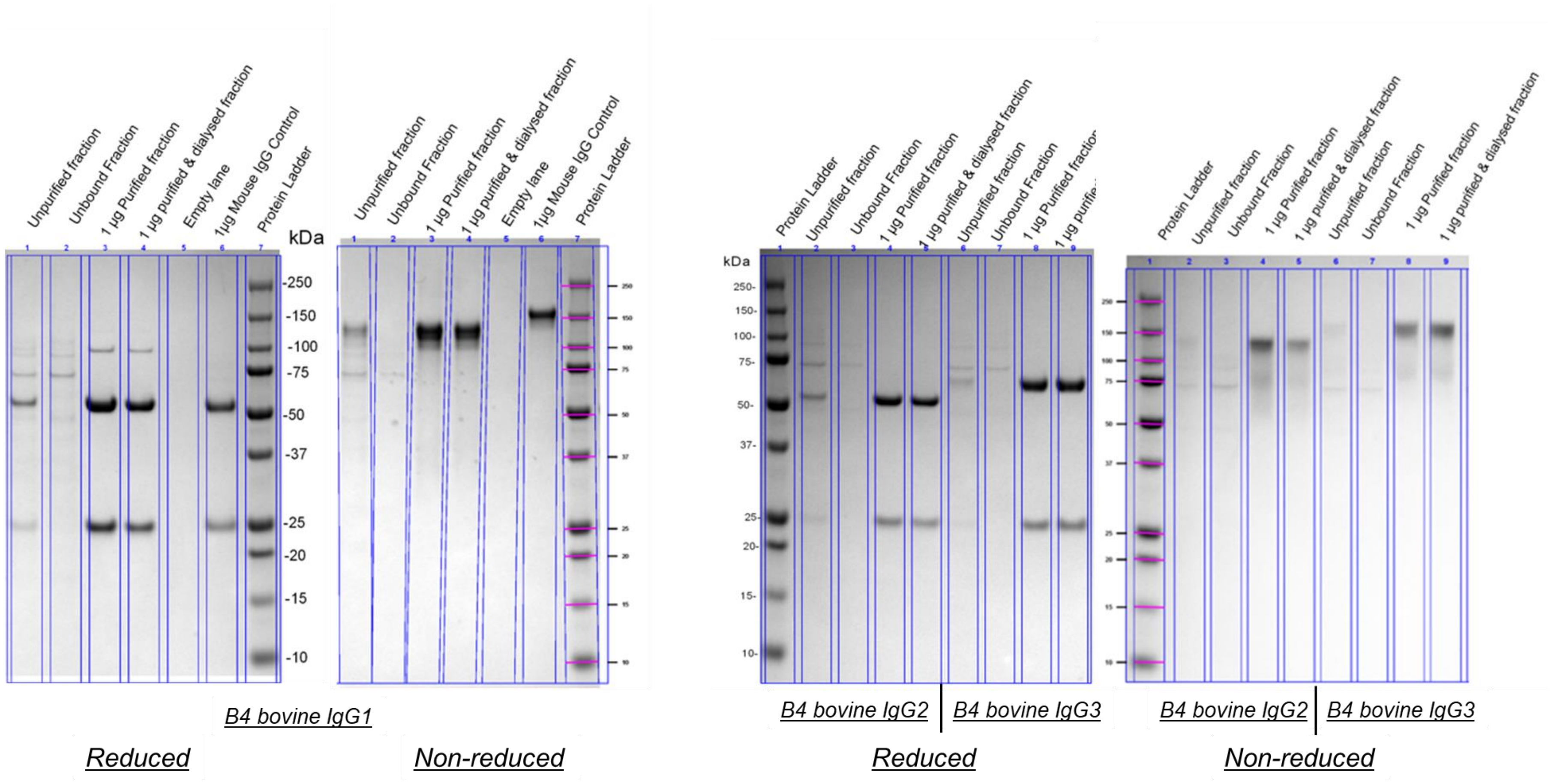

Supplementary Figure 2. SDS-PAGE (4-12% Bis-Tris gel) analysis of purified recombinant chimeric B4 mAb expressing bovine IgG1, IgG2 and IgG3 and murine IgG2a Fc regions.

Supplementary Table 1. Total protein yields for recombinant chimeric monoclonal antibodies expressing bovine IgG Fc regions.

Fab region	Fc region	Yield (mg/L)
pb27	IgG1	46
	IgG2	48
	IgG3	46
B4	IgG1	51
	IgG2	31
	IgG3	29

>bovFcγR1A

MPMGSLQPLATLYLLGMLVASVLAVDPTKAVITLKPPWVSVFQEENVTLLCEGPHRPGDTATQWFLNGTAI
KTLAPRYSINSATFDDSGEYKCQTGLSMLSDPVQLEIHSDWLLLQVTSRVFTEGDPLALRCHAWKNMPVYK
MLFYKDGKPFRFSSQDSEFTILQTNLSHNGIYHCSGERRRRYTSAGVSITIKELFPAPVLRTSFSSPHQEG
NLVNLSCETKLPSEKPGQQLYFSFYVGNKTLISRTTSSEYQTFIAKKEDRRLYWCEAATGDGNLIKRSPEL
ELPVLGLQSTTP**VWFH**GLNDIFEAQKIEWHEHHHHHH*

>bovFcγR2A

MGIPSFLAFPAARRNRAHCTPWHPWGHMLLWTALPFLAPVPGKCADLPKAVVSIQPAWINVLREDHVTLMC
QGTSFSAGNLTTWFHNGSSIHTQKQPSYSFRAGSNDGGSYRCQREQTSLSDPVHLDVISDWLLLQTPSLVF
QEGEPIMLRCHSWRNQPLNKITFYQDGKSKTFSYQRTNFSIPRANLSHRGQYHCTAFIGKMLHSSQPVNIT
VQDGNEGPAVPLIFSGLNDIFEAQKIEWHEHHHHHH*

>bovFcγR3A

MPMGSLQPLATLYLLGMLVASVLAADPSKAVVLLDPQWNHVLTNDRVTLKCQGDYPVEDNSTKWWHNGTLI
SSQTPSYFIADVKVQDSGEYKCQTGLSAPSDPVKLEVHVGWLLLQVAQRVVNVGKPIRLKCHSWKKTPVAK
VQYFRNGRGKKYSHGNSDFHIPEAKLEHSGSYFCRGIIGSKNESSESVQITVQAPETLQTVSSFFLP**WHQ**G
LNDIFEAQKIEWHEHHHHHH*

>bovFcγ2R

MGILPSPGMPALLSLVSLLSVLLMGCVAGTFPKPIIWAEPSSVVPLGSSVTILCQGPPNTKSFSLNKEGDS
TPWNIHPSLEPWDKANFFISNVREQQAGRYHCSHFIGVNWSEPSEPLDLLVAGEEPAGRLRDRPSLSVRPS
PSVALGENVTLLCQSGNRTDTFLLSKEGAAHRPLRLRSQDQDGWYQAEFSLSPVTSAHGGTYRCYRSLSTN
PYLLSQPSEPLALLVADYTMQN**LI**GLNDIFEAQKIEWHEHHHHHH*

Signal peptides

Bovine FcγR extracellular domains

Transmembrane region motif used as linker

Avi biotinylation tag

His6 tag

Supplementary Figure 3. Bovine Fc gamma receptor constructs – peptide sequences used for constructs used in Figure 2.

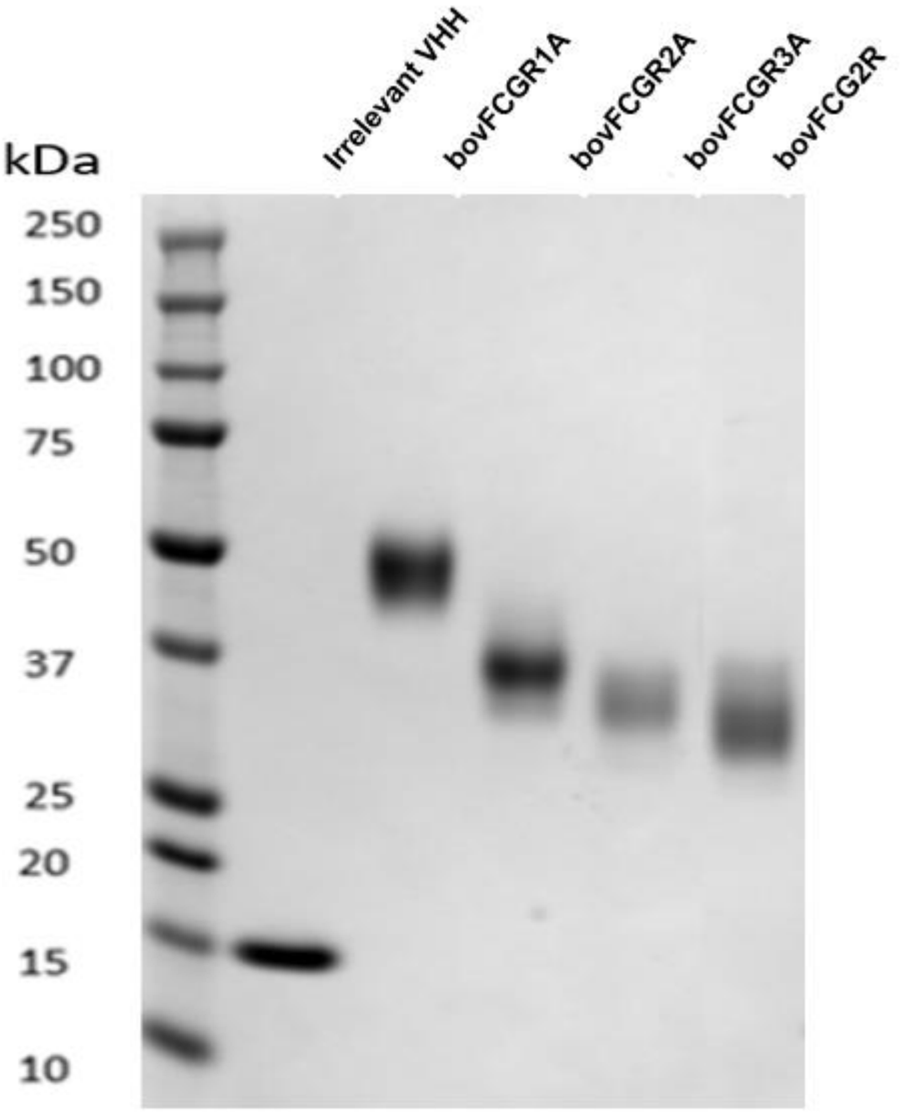

Supplementary Figure 4. SDS-PAGE analysis of purified recombinant soluble bovine Fc gamma receptor constructs. 1 µg protein loaded per well in reducing conditions. Predicted molecular weights (kDa): FCγR1A - 36.36; FCγR2A - 28.22; FCγR3A - 26.23; and FCγ2R - 25.66.

Supplementary Table 2. Total protein yields for recombinant soluble bovine Fc gamma receptors.

**Protein**	**Yield (mg/L)**
FCγR1A	9
FCγR2A	5
FCγR3A	11
FCγ2R	50

Supplementary Table 3. Antibodies used:

**Antigen**	**Label**	**mAb Clone**	**Supplier**
CD3	PerCP-Cy5.5 (Lightning-Link labelled)	MM1A	Bio-Rad
CD11b	A647	CC126	Bio-Rad
CD14 (human)	APC-Cy7	M5E2	BioLegend
CD16 (human) (FcgRIII)	APC-Cy7 (Lightning-Link labelled)	KD1	Bio-Rad
CD32 (FcgRII)	FITC	CCG36	Bio-Rad
CD40	FITC	IL-A156	Bio-Rad
CD41/CD61	PE-Cy7 (Lightning-Link labelled)	IVA30	Thermo Fisher
CD107a	APC	9f6E2/EC2	Gift from Timothy Connelley, Roslin Institute, UK
CD172a	unlabeled	CC149	Bio-Rad
NKp46 (CD335)	PE	AKS1	Bio-Rad
IgG (H&L)	biotin	Polyclonal	Thermo Fisher
IgG1/2/3Pan IgG	PerCP-Cy5.5 (Lightning-Link labelled)	LS-C814448	Stratech

Supplementary Table 4. Primers used for qPCR:

**Target mRNA**	**Primer sequence**	**Reference**
FcγRI / CD64	Fwd: 5’-TGAGGTGTCATGCATGGAAG-3’<br>Rev: 5’- AGATACTCCTGCCGATGTGT-3’	Designed
FcγRII / CD32A	Fwd: 5’-TCCAGCAGTTCCACTCATCT-3’<br>Rev: 5’-AGGGTCATTTGTCCTGAGGG-3’	Designed
FcγRIII / CD16	Fwd: 5’-TCTCATCTCAAGCCAGACCC-3’<br>Rev: 5’-CCCACATTTACCACCCGTTG-3’	Designed
Fcγ2R / FCG2R	Fwd: 5’-AGTCTGAACAAAGAGGGCGA-3’<br>Rev: 5’-GCTCTTCTCCTGCCACCA-3’	Designed
GAPDH	Fwd: 5’-GGTCGGAGTGAACGGATTTG-3’<br>Rev: 5’-TGGCAACGATGTCCACTTTG-3’	Ref 42

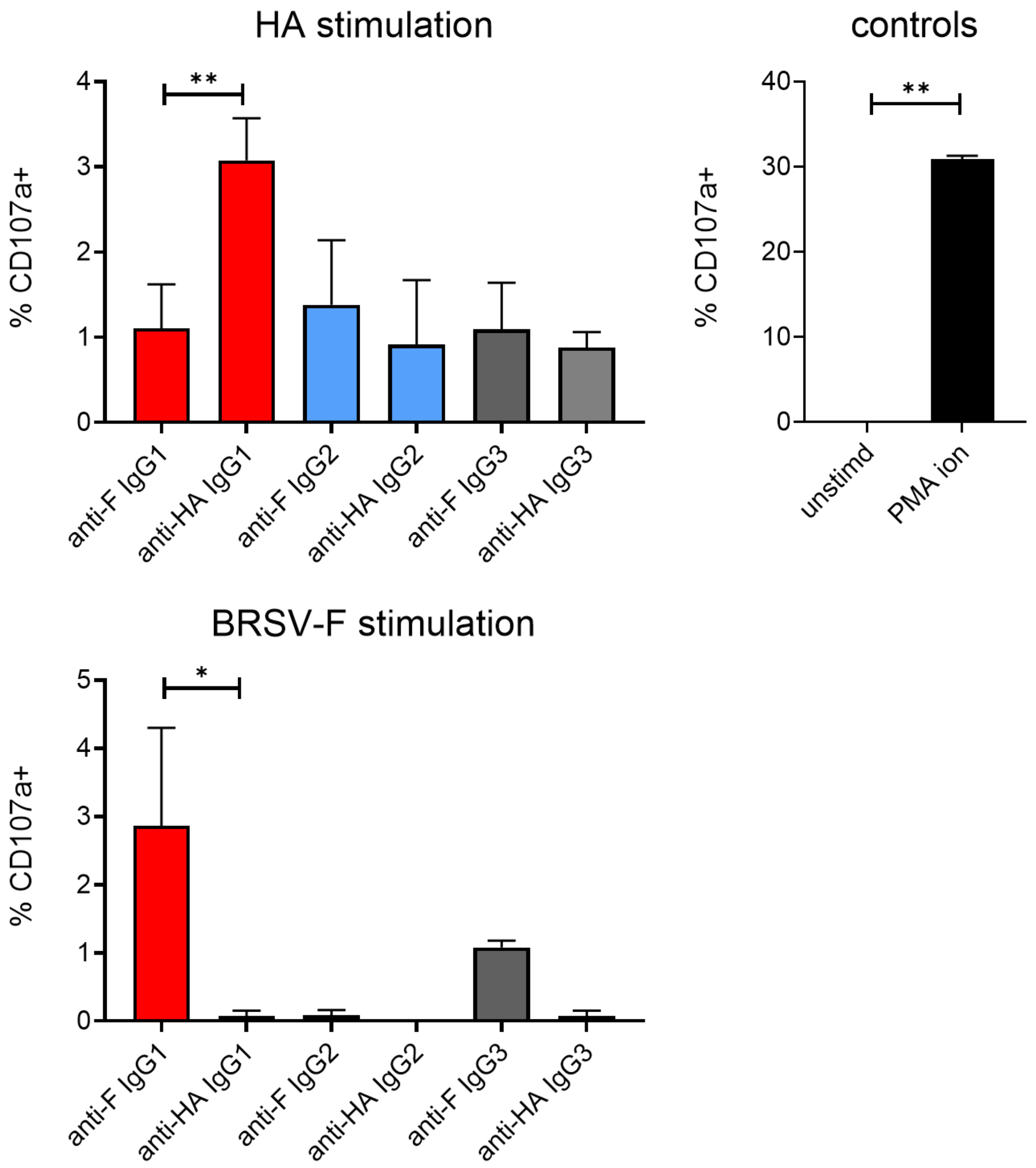

Supplementary Figure 5. Cross-over experiment confirms IgG1 mAbs induce NK cell degranulation. NK cell stimulation was performed as in Fig 7, with cell stimulation using plate-bound HA or BRSV-F protein as indicated. Anti-BRSV mAbs were used as controls throughout the study. Data shown are means ± SEM from two independent experiments with background levels of CD107a subtracted. Significant differences between groups are indicated (one-way ANOVA).
